# A Systematic Review of Controlled Trials: Can Patient Adherence to Antibiotics Be Improved Through Pharmaceutical Communication-Based Interventions?

**DOI:** 10.3390/pharmacy12060178

**Published:** 2024-11-26

**Authors:** Carla Pires

**Affiliations:** CBIOS-Universidade Lusófona Research Center for Biosciences and Health Technologies, Campo Grande, 376, 1749-024 Lisbon, Portugal; p5558@ulusofona.pt

**Keywords:** pharmacist, antibiotics, patient adherence, communication-based interventions

## Abstract

Background: Patient adherence to antibiotics is vital to ensure treatment efficiency. Objective: To evaluate the impact of pharmacist communication-based interventions on patients’ adherence to antibiotics. Methods: A systematic review following the Preferred Reporting Items for Systematic Reviews and Meta-Analyses for systematic review (PRISMA) checklist and flow diagram. Controlled trials were included. Databases: PubMed, Cochrane Library, SciELO, and Google Scholar. Quality, risk of bias, and confidence in cumulative evidence were evaluated. Results: Twenty-one trials were selected, with better patient adherence for the intervention than the control group. However, statistically significant differences were only found in two-thirds of these trials. The use of educational leaflets, personalized delivery of antibiotics, follow-up measures, and structured counseling were among the most impactful and significant interventions. The fact that community and/or hospital pharmacists were required to intervene in both groups (e.g., intervention vs. control/usual care) may explain that statistically significant differences were not achieved in all trials. Moderate quality issues and/or risk of bias were detected in some of the evaluated trials. The cumulative evidence was classified as high to moderate, which was considered acceptable. Conclusion: It seems that more intense and structured pharmacist interventions can improve patient adherence to antibiotics.

## 1. Introduction

Pharmacists should be available daily to patients, at both hospitals and/or community pharmacies. Pharmacists should ensure effective therapy management (e.g., patient adherence, medication-related outcomes, pharmacovigilance, and reconciliation of therapy), in addition to preparing, obtaining, storing, securing, distributing, administering, dispensing, and disposing of medicinal products, among others [1,2].

Particularly, the management of antibiotic therapy by pharmacists seems relevant to ensuring patient adherence to antibiotics. In the European Union, pharmacists are required by law (i) to dispense prescribed antimicrobials, (ii) to ensure the comprehension of patients about the dosage, frequency, and duration of treatment, (iii) to actively participate in the disposal of non-used antibiotics, (iv) to handle notifications of drug-adverse reactions, (v) to provide information and clarify doubts about the precautions, contraindications, and interactions of antimicrobials, and (vi) to participate in public health programs/campaigns about the rational use of antibiotics [2].

According to the World Health Organization (WHO) key facts, “the misuse and overuse of antimicrobials in humans, animals and plants are the main drivers in the development of drug-resistant pathogens”, with the possible appearance of antimicrobial resistances (AMRs). AMRs occur “when bacteria, viruses, fungi and parasites change over time and no longer respond to antimicrobial medicines”, and they are estimated to have been directly responsible for 1.27 million deaths at a global level in 2019 [3]. Decreased patient adherence to antibiotics can favor the appearance of AMRs, as well as a reduction in treatment efficacy [2,3].

Patient adherence to antibiotics is related to the completion of an antibiotic course as prescribed (not self-medicating) [4]. Compliance and adherence are interrelated concepts. For instance, compliance can be defined as “the extent to which the patient’s behavior matches the prescriber’s recommendations”, and adherence can refer “to a process, in which the appropriate treatment is decided after a proper discussion with the patient” [5].

The education of patients by pharmacists can support ameliorated patient adherence to antibiotics, symptom assessment, dispensing first-line antibiotics, and decreasing the OTC dispensing of antibiotics, consequently contributing to minimizing the risk of AMRs and ensuring the efficacy of treatment according to findings from some studies [4,6]. However, the systematic review and metanalysis of Lambert et al. (2022) concluded that “adherence to antibiotics did not significantly increase after pharmacist-led interventions”, based on the findings of 9 out of 17 selected studies [6]. Thus, the following research questions were defined:

- What is the impact (positive or negative) of pharmacist communication-based interventions on patients’ adherence to antibiotics in the selected studies?
- What are the types of pharmacist communication-based interventions to improve antibiotic adherence adopted in community and hospital pharmacies in the selected studies?

Additionally, primary and secondary objectives were defined, as follows:

- To evaluate the impact of pharmacist communication-based interventions on patients’ adherence to antibiotics in the selected studies (primary objective).
- To identify different types of pharmacist communication-based interventions to improve antibiotic adherence in the selected studies (secondary objective).

## 2. Materials and Methods

### 2.1. Ethics Statement

Ethical approval was not required since the present work is a systematic review.

### 2.2. Type of Study, Previous Registration, and Published Protocol

A systematic review was conducted following the requirements of the JBI guidance [7] and reported according to the PRISMA checklist and flow diagram [8]. The full version of the PRISMA-P checklist applied to the present systematic review can be consulted in a previous publication [9]. The detailed protocol of the present systematic review is registered in OSF Registries (registration number: osf.io/sba2z).

### 2.3. Population, Intervention, Comparison, and Outcome (PICO)

The PICO model was used to support the formulation of objectives and develop the search strategy [10] as follows: population (pharmacists from a community or hospital pharmacy); intervention (any pharmacist communication-based interventions, such as patient counseling/education, interviews, workshops, the provision of written information, or other); comparison (controlled trial: control group vs. group of patients enrolled in a pharmacist communication-based intervention), and outcomes (positive or negative impact on patients’ antibiotic adherence).

### 2.4. Inclusion and Exclusion Criteria

Inclusion criteria: Controlled trials aimed at evaluating the impact of pharmacist communication-based intervention on patients’ antibiotic adherence in a community or a hospital pharmacy (control group vs. any communication-based pharmacist intervention group). Patients had to take at least one antibiotic. Only original research was included. Exclusion criteria: Published papers not written in English, Spanish, Portuguese, French, or Italian. Commentaries, reviews, qualitative studies, letters to editors, and preprints were also excluded.

### 2.5. Screened Databases/Searched Resources, Keywords, and MeSH Terms

Synonyms, related keywords (e.g., adherence and compliance) and/or MeSH terms (Table 1) were selected to ensure the inclusion of a broader number of studies than those identified in previously published reviews related to the present topic, as well as to identify the most comprehensive findings/studies [6,11]. PubMed was selected because it is an optimal tool in biomedical electronic research. SciELO was selected to ensure the inclusion of papers in Spanish and Portuguese. Cochrane Library was selected to ensure the detection of previous reviews related to the present topic. Google Scholar covers most scientific fields and comprises around 389 million records, i.e., a much higher number of records than other databases/resources [12].

### 2.6. Dates of Searches per Searched Database/Resource and Covered Timeframe

The searches were conducted without a time limit. Searches were conducted in January 2024. The searches were, respectively, carried out as follows: PubMed (6-1-2024), Cochrane Library (7-1-2024), SciELO (8-1-2024), and Google Scholar (11-1-2024). PDFs of all searches were archived for later consultation (if necessary).

### 2.7. Screening Process and Data Collection

The screening process and data collection were conducted by just one researcher, as follows (steps 1–4).

Step 1: (i) Search of the strings of keywords per each database/resource; (ii) exclusion of duplicated studies; (iii) titles and abstracts were read; (iv) selected papers based on title/abstract were archived; and (v) consultation of the full version of all studies/trials before validating their exclusion/inclusion. Motives of exclusion were annotated.

Step 2: Reassessment of the selected studies/trials to validate their inclusion/exclusion. Motives of exclusion were annotated.

Step 3: a tabular format was used to register the extracted data.

Step 4: Steps 1 to 3 were repeated because just one researcher carried out the present systematic review; i.e., steps 1 to 3 were repeated through two separate procedures with the aim of identifying eventual discrepancies. Discrepancies were not identified. 

### 2.8. Collected Variables and Data Synthesis

The data collected were registered in a tabular format (see Table 2). The collected data were double-checked. The study findings were synthetized based on a narrative synthesis with reference to the quantitative/qualitative collected data.

### 2.9. Quality Assessment of the Selected Studies

The National Heart, Lung, and Blood Institute (NHLBI) quality assessment tool for Quality Assessment of Controlled Intervention Studies was applied [34]. Question 4 of the NHLBI of the quality assessment tool, “4. Were study participants and providers blinded to treatment group assignment?” was excluded because it is impossible to keep community or hospital pharmacists blind regarding a certain intervention. The JCR impact factor of the journal of the selected studies/trials was quantified because papers from journals with a JCR impact factor are peer-reviewed. 

### 2.10. Evaluation of the Risk of Bias and Confidence in Cumulative Evidence

A more simplified methodology based on the original tool (i.e., Rob2 for randomized trials) was adopted in the evaluation of the risk of bias of the selected randomized trials because the detailed protocols of the selected trials were not fully available in the published papers, and the selected trials evaluated a social intervention (i.e., the impact of a pharmacist communication-based intervention on patient adherence to antibiotics) (not the administration of a medicine in a clinical trial). The specifically evaluated variables were as follows [35,36]:

- “Random sequence generation”;
- “Allocation concealment”;
- “Blinding of outcome assessment”;
- “Not incomplete outcome data”;
- “Not selective reporting”;
- “Not other bias”.

Particularly, the option “blinding of participants and/or personnel” was not considered in the present evaluation because it is not applicable in the present social evaluation (i.e., pharmacists are required to know about the intervention). 

The evaluation of the risk of bias for the non-randomized selected studies was based on the ROBINS-I tool, with the following evaluations [37]:

- Pre-intervention (bias due to confounding and bias in the selection of participants for the study);
- During the intervention (bias in classification of interventions);
- Post-intervention (bias due to deviations from intended interventions, bias due to missing data, bias in measurement of outcomes, and bias in the selection of the reported result).

### 2.11. Confidence in Cumulative Evidence: GRADE-CERQual

The GRADE-CERQual for qualitative studies was adopted to evaluate the confidence in cumulative evidence since the adopted statistical methodologies were variable between the selected studies, and the magnitude of the effects was not presented in all cases. Overall, four elements were evaluated: (i) methodological limitations, (ii) coherence, (iii) adequacy of data, and (iv) relevance. The findings were rated for confidence as follows: “Very low”, “Low”, “Moderate”, and “High” [38]. 

GRADE for quantitative studies was not adopted to evaluate the confidence in cumulative evidence because the study methodologies of the selected trials were too heterogeneous. The selected trials were based on different types of pharmaceutical interventions (e.g., interviews, phone calls, different educational interventions, etc.) and were not clinical trials. For instance, a proper evaluation of inconsistency, indirectness, or imprecision was not considered viable because the adopted methodologies of interventions between the selected trials were different. Thus, the evaluated effect sizes were not comparable, and GRADE for quantitative studies was not applicable.

### 2.12. Motives for Not Carrying out a Metanalysis

A meta-analysis to support a quantitative analysis was not carried out because the measures of effect were not presented in all the selected studies; the selected trials were not sufficiently homogeneous in terms of their design and comparators, and the adopted statistical methodologies of the selected trials were heterogeneous (for additional information, please see the subsection on limitations) [10]. 

## 3. Results

### 3.1. PRISMA 2020 Flow Diagram

**Figure 1:**
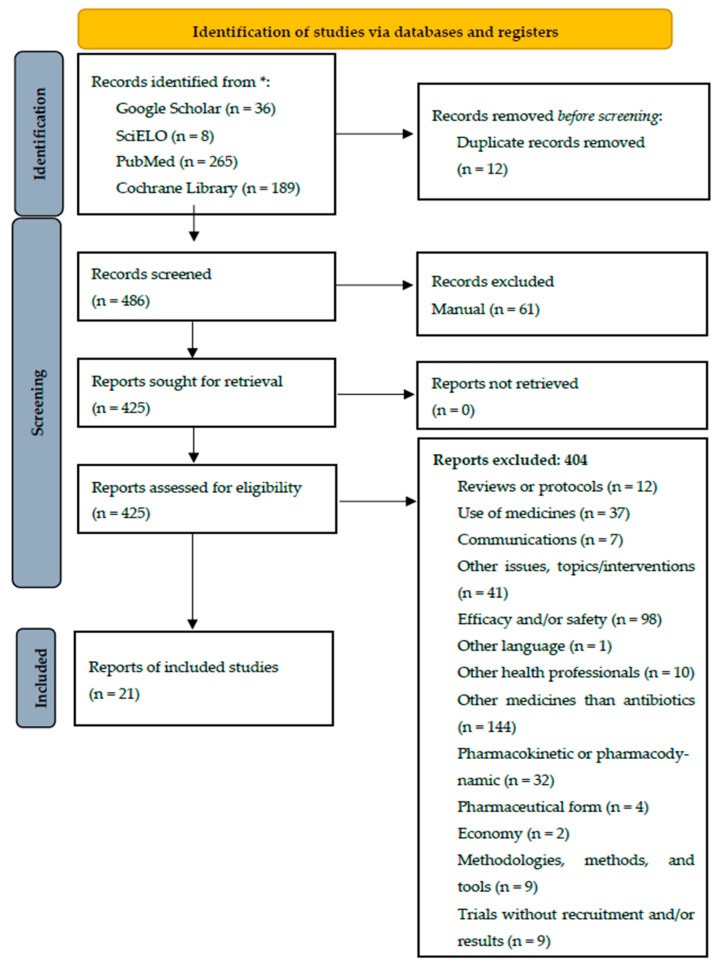
Identification of studies via databases/resources and registers for the present systematic review. * Number of records identified from each database/resource.

Overall, 21 studies/trials were selected. The identification of studies via databases/resources and registers is represented in Figure 1, which followed the PRISMA 2020 flow diagram for new systematic reviews [8,39].

### 3.2. Main Findings: Collected Variables

The main findings of the selected studies/trials are presented in Table 2.

Globally, the impact of pharmacist intervention was positive on patients’ adherence to antibiotics in the analyzed studies. Statistically significant differences between the control and intervention groups were not found in one-third of the selected trials (7; 33.3% of the 21 selected trials) [16,17,18,23,25,26,30]. However, the findings/proportions were quantitatively better in the intervention group than in the control group of these seven trials [16,17,18,23,25,26,30].

### 3.3. Different Types of Pharmacist Communication-Based Interventions to Improve Antibiotic Adherence

The adopted methodologies for pharmacists to improve patient adherence were conveniently grouped by type of intervention and/or treatment interventions in five groups (i.e., according to five descriptors) as follows: (i) visual aid [13,26]; (ii) the dispensation of a syringe for correct dosing or personalized delivery (per unit) [14,19]; (iii) using both oral and written information in the intervention group [17,20,23,25,32,33]; (iv) oral education-based interventions (excluding counseling in the case of *H. pylori* treatment) [18,22,24,27,28,29,30,31,32,33]; and (v) counseling in the case of *H. pylori* treatment [15,16,21] since these therapeutics usually involve multiple medicines, which may complicate patient adherence. These descriptors were used to classify the selected studies (Table 2) and carry out a more comprehensive discussion.

### 3.4. Quality Assessment of the Selected Studies

Two out of the twenty-one selected studies/trials were not included in the quality assessment: an abstract [28] and a pilot study [33]. Overall, questions 9–11 and 13 from the NHLBI assessment tool were 100% compliant for all the selected studies [34] (Table 3).

The results from questions 1 and 6–8 (compliance > 80%) of the NHLBI assessment tool were classified as potentially acceptable [34] because these trials were based on social-work interventions (e.g., they were not clinical trials specifically designed to evaluate the safety and efficacy of a certain medicine). Conversely, the results from questions with <80% compliance (i.e., questions 2–3, 5, 12, and 14 from the NHLBI assessment tool) were classified as potential quality issues. Detailed, full reports of the selected papers were not identified. Moreover, authors from the selected trials were not contacted to check whether the evaluations from these questions (i.e., questions 2–3, 5, 12, and 14) were (or were not) implemented and/or carried out. 

### 3.5. Risk of Bias

Two of the selected trials were not evaluated in the assessment of a risk of bias: an abstract [28] and a pilot study [33]. The only eventually identified bias was related to the selection of participants because the randomization methodology was not reported in these two trials [24,25]. The results of the selected randomized trials (n = 17) were as follows: “Incomplete outcome data” or “elective reporting” were not detected (100% trials were classified as compliant, i.e., no risk of bias) and the % of eventual risk of bias (non-conformities) were as follows: “not reporting blinding of outcome assessment” (76.5%); “not reporting allocation concealment” (70.6%); “not reporting random sequence generation” (47.1%); and risk of “other bias, i.e., not exhaustively describing or not describing at all the routine pharmaceutical intervention” (35.3%).

### 3.6. Confidence in Cumulative Evidence: GRADE-CERQual

The findings concerning confidence in cumulative evidence through GRADE-CERQual are presented in Table 4.

## 4. Discussion

In general, positive outcomes were achieved in all the selected studies (n = 21) (i.e., better results in the intervention group than in the control group), with significant differences in two-thirds of the selected studies and non-significant differences in one-third of the selected studies. Statistically significant differences could not have been achieved because of the heterogeneity of the methodologies of the selected studies, differences in patient populations, variability in intervention protocols, limited follow-up durations, different practices between hospital and community pharmacists, the possibility of different practices between different regions, and the fact that pharmacists were required to intervene in both groups (e.g., intervention vs. control or usual care), which may have been due to deontological motives. Overall, usual care was provided in the control group vs. an intervention group (i.e., usual care plus an additional intervention, involving a reinforced pharmacist intervention) (Table 2). It seems that usual care, or routine pharmaceutical practice, can be optimized through a more intense and structured pharmacist intervention.

The present systematic review is the most representative work on the present topic (21 analyzed trials) as far as is known. However, the previous systematic review and metanalysis of Lambert et al. (2022) found that “adherence to antibiotics did not significantly increase after pharmacist-led interventions” through findings that were based on only 9 out of the 17 selected studies [6]. The objectives of the systematic review and metanalysis of Lambert et al. (2022) were “to assess the effects of community pharmacist-led interventions to optimize the use of antibiotics and identify which interventions are most effective” [6], i.e., broader objectives than the objectives of the present systematic review. It is important to note that, of the 9 (out of 17) studies identified by Lambert et al. specifically concerning the impact of pharmacist-led interventions on patients’ adherence to antibiotics, 8 were also included in the present systematic review [17,20,23,25,26,29,32,33]. One of these nine studies was not included in the present systematic review because it was impossible to retrieve.

### 4.1. Different Types of Pharmacist Communication-Based Interventions to Improve Antibiotic Adherence

#### 4.1.1. Visual Aid

A visual aid seems to be a simple and accessible methodology to improve patient adherence to antibiotics, such as in the case of nonliterate patients [13]. Health information materials with pictures improved patient knowledge/understanding, and recall can support better patient adherence to medicines (e.g., pictograms or other visual aids) [13,26,40].

#### 4.1.2. Dispensation of a Syringe for Correct Dosing or Personalized Delivery (Per Unit)

Particularly, the use of oral syringes facilitated the measurement and administration of liquid medicines by caregivers, and it minimized the exposure to any potentially unpleasant smell [14,41]. The demonstration on how to carry out correct dosing using a syringe and the confirmation of patients’ understanding of this procedure can reduce dosing mistakes [42].

The personalized delivery of antibiotics vs. standard packaging also produced a strong positive impact on patient adherence [19]. Advantageously, antibiotic waste can be reduced through the personalized delivery of antibiotics [19,43]. These facts were also verified in other studies. For instance, caregivers better adhered to the use of pre-packed tablets than chloroquine syrup, with only 20% of the caregivers using an accurate 5 mL measure for children diagnosed with malaria (aged 0–5 years) [43].

#### 4.1.3. Oral Plus Written Information

The use of both oral and written information, such as a package insert for medicines, by patients may have a positive, statistically significant impact on adherence to medication/antibiotics, according to their perceptions [17,20,23,25,32,33]. It seems that leaflets/written information can be successfully dispensed to support pharmacists’ structured counseling and, consequently, enhance patients’ adherence to antibiotics. However, statistically significant findings were not achieved in all the studies [44], which may be explained by the use of too-complex materials or non-pre-tested written information.

#### 4.1.4. Oral Interventions

Pharmaceutical care is defined as a “patient-centred pharmacist activity to improve medicines management by patients and encompasses a variety of specific services” [45]. Structured interventions (e.g., oral interventions) are known for producing positive patient health outcomes, such as resolving drug-related problems or improving medicine adherence [46]. The oral pharmaceutical interventions of the selected trials adopted very heterogeneous methodologies, as follows: motivational interviews to address negative health behaviors, such as adherence [18]; reinforced education about the correct use of antibiotics [22,25,28,29]; counseling about antibiotics, followed by a phone call [27]; the evaluation of the intention to take a certain antibiotic (e.g., theory of planned behavior) [30]; and the use of a model/tool to support a pharmacist intervention, followed by a follow-up phone call [31]. Phone calls can be used to monitor the safety and efficacy of antibiotic treatment, such as adherence or the eventual occurrence of side effects. It seems that the structured education of patients by a pharmacist is a successful methodology to improve patient adherence to antibiotics. Structured education can be supported through a tool to check and orient a pharmaceutical consultation [31], for example, if integrated in the scope of a pharmaceutical care program.

#### 4.1.5. Counseling in the Case of *Helicobacter pylori* Treatment

Helicobacter pylori infection is related to diverse upper gastrointestinal diseases, such as chronic gastritis, peptic ulcer, or gastric cancer. Half of the world population is estimated to carry *H. pylori*, with the main therapy involving the use of three or four medicines (e.g., amoxicillin, furazolidone, clarithromycin, levofloxacin, metronidazole, and a proton pump inhibitor). The enhancement of patient medication adherence, reduction in adverse drug reactions, and improvement in *H. pylori* eradication rates can be advanced with statistical significance through pharmacists’ intervention [15,16,21,47]. The adoption of structured counseling, the implementation of an educational program, a follow-up phone call, or the provision of additional counseling successfully strengthened patient adherence [15,16,21], which may be explained because of the complexity of *H. pylori* treatment (e.g., the simultaneous use of three or four medicines and different drug regimens).

### 4.2. Comparison Between Different Types of Pharmacist Communication-Based Interventions to Improve Antibiotic Adherence

All pharmacist interventions ameliorated patient adherence (significant differences in two-thirds of the selected studies and non-significant differences in one-third of the selected studies), although it is not possible to conclude about the best adopted methodology or to compare findings from different research since the study designs, statistical methodologies (e.g., Tukey and Fisher’s LSD multiple-comparison test, Student’s t-test, Fisher’s exact test, chi-square test, etc.) [13,15,18], methods, interventions, settings, etc. were different and very heterogeneous across the selected studies (n = 21) (Table 2). For instance, the concept of adherence and compliance was applied with the same meaning in some of the selected studies, and the methodologies for measuring patient adherence were heterogeneous between the selected studies, such as patient self-assessment (e.g., phone calls or presential interviews at a pharmacy), pill counts, the application of formulas, the Morisky–Green test, or mixtures of these methodologies (Table 2). Ideally, the application of more than one methodology is recommended to evaluate patient adherence since patients’ self-reporting of adherence may be related to imprecisions (e.g., memory issues) or since pill counts per se are not enough to check adherence because patients may not take antibiotic pills correctly (e.g., duplication of pill intake).

### 4.3. Quality Assessment and Risk of Bias of the Selected Studies

The interpretation of the findings of the present systematic review may have been affected by some potential quality issues and/or the risk of study bias. For instance, the sample size calculation (e.g., with at least 80% power) and/or “an intention-to-treat analysis” were not reported in an expressive number of trials, which may have affected the quality of the study findings. Likewise, not exhaustively describing (or not describing at all) routine pharmaceutical interventions may have been related to a negative impact on study reproducibility, as well as on studies’ comparability with other, similar studies. In contrast, not carrying out “blinding of outcome assessment” may not have produced major quality issues and/or a risk of bias because, in most of the selected studies, pharmacists were required to ask closed or semi-closed questions to assess adherence (i.e., outcome assessment), as well as to collect and record patients’ replies.

In general, “allocation concealment” (researchers do “not know in advance or cannot guess accurately, to what group the next person eligible for randomization will be assigned”) was not reported in the selected studies, although nowadays, computer-generated randomization/random sequence generation is one of the most common randomization methodologies. It is important to note that only the published papers were assessed (not the full protocol studies) for both randomized and non-randomized trials. Thus, some quality evaluations may not have been precise since the full versions of the studies’ protocols were not analyzed. Most trials were published in journals with a JCR impact factor higher than two, which is necessarily related to peer-reviewed journals.

### 4.4. Limitations

It was not possible to carry out a meta-analysis regarding, some of the selected studiesÂ´ use of the following qualities: “different methods to define exposure and/or outcome”; “different study designs were used”; “different analyses and methods were applied to generate the estimates”; or “there were variation in populations included across different studies; studies differ by their quality/risk of bias” [10,48,49]. Additionally, to carry out a meta-analysis, “a summary statistic is calculated for each study, to describe the observed intervention effect in the same way for every study and the summary statistic may be a risk ratio if the data are dichotomous, or a difference between means if the data are continuous” [48], although measures of effect were not reported in all the selected studies. The potentially detected study quality may have affected the accuracy of the findings of the present systematic review. Pharmacists’ practices and regulations, as well as practices and interactions with hospital and community pharmacists, may differ across the selected trials, given that the trials were carried out in different countries. 

#### Limitations of Methods

It is important to notice that applying a clinical studies mindset to a social phenomenon can be epistemologically and ontologically misleading. Thus, the adopted methodologies in the present study, such as the NHLBI Quality Assessment of Controlled Intervention Studies, a simplification of Rob2 for randomized trials, or GRADE-CERQual can also be related to some constraints. Positively, the adopted methodology respects all the requirements defined for a narrative summary of evidence as follows: group studies (step 1); following the same synthesis consistently (step 2); reporting findings clearly (step 3); and discussing findings objectively (step 4) [49].

The adopted methodological tools were applied twice by just one researcher. The number of screened databases may have been limited since Scopus and Web of Science (paid databases covering most scientific fields) were not browsed. However, the number of selected studies in the present systematic review was broader than a previous review and meta-analysis about a related topic (21 in the present systematic review vs. 17 in the systematic review and meta-analysis by Lambert et al., with only 9 out 17 specifically covering patients’ adhesion to antibiotics) [6]. CiteScore could have been used instead of the JCR impact factor, although CiteScore and the JCR impact factor metrics seem to be positively correlated [50]. Only published papers were evaluated (not the full protocols), which may have introduced some inconsistencies in the performed evaluations of quality or risk of bias.

### 4.5. Strengths

As far as is known, this is the first systematic review to have specifically explored the impact of hospital or community pharmacists’ communication-based interventions on patient adherence to antibiotics. The selected studies involved an expressive number of participants, which may have contributed to higher research accuracy. The studies were conducted in different regions, which is likely to support an easier extrapolation of data. Systematic reviews with (or without) meta-analyses are likely to provide appropriate and a high-level quality of evidence [51]. The findings of the present systematic review are congruent with data from previous related studies (e.g., improvement in patient adherence, knowledge of medications, quality of life, physical function, and symptoms in patients receiving a medication-adherence intervention or the relationship between the healthcare and patient, such as the provision of patient education, training, and follow-up, and the time availability of consultation, among others, supporting improved patient adherence) [52,53].

## 5. Conclusions

Patient adherence to antibiotics improved with more intense pharmacist communication-based interventions when compared to the routine/regular practice at hospitals or community pharmacies in the analyzed studies. However, statistically significant findings between usual care vs. intensive care were not achieved in all the selected trials. Thus, a more structured and proactive pharmacist intervention is likely to significantly support and improve patient adherence to antibiotics.

Pharmacists’ interventions to improve antibiotic adherence were very heterogeneous, such as oral and/or written education-based interventions, intensive counseling, interviews, visual aids (e.g., pictograms), follow-up phone calls, or personalized delivery (i.e., the dispensation of an exact number of pills). Finally, reinforced pharmaceutical interventions seem to be especially useful for patients with low literacy and in more complex therapeutic regimes, such as *H. pylori* treatment.

## Figures and Tables

**Table 1:** Search strategy per searched resource.

Database/Searched Resource, Activated Fields, and Link	Strings of Keywords and/or MesH Terms
PubMed * (all fields) https://pubmed.ncbi.nlm.nih.gov/advanced/ (access date: 6 January 2024)	(antibiotic or antimicrobial or anti-bacterial or antibacterial) and (adherence or compliance) and pharmacy (antibiotic or antimicrobial or anti-bacterial or antibacterial) and “patient adherence” and pharmacy (antibiotic or antimicrobial or anti-bacterial or antibacterial) and “patient compliance” and pharmacy (antibiotic or antimicrobial or anti-bacterial or antibacterial) and (adherence or compliance) and pharmacist (antibiotic or antimicrobial or anti-bacterial or antibacterial) and “patient adherence” and pharmacist (antibiotic or antimicrobial or anti-bacterial or antibacterial) and “patient compliance” and pharmacist
Cochrane Library (Title Abstract Keyword) https://www.cochranelibrary.com/ (access date: 7 January 2024)	The same strings of keywords or MesH terms used for PubMed
SciELO (all fields) https://www.scielo.org/ (access date: 8 January 2024)	The same strings of keywords or MesH terms used for PubMed
Google Scholar (all fields) https://scholar.google.com/ (access date: 11 January 2024)	“patient adherence” and (antibiotic or antimicrobial or anti-bacterial or antibacterial) and “community pharmacy” and pharmacist and trial “patient adherence” and (antibiotic or antimicrobial or anti-bacterial or antibacterial) and “hospital pharmacy” and pharmacist and trial “patient compliance” and (antibiotic or antimicrobial or anti-bacterial or antibacterial) and “community pharmacy” and pharmacist and trial “patient compliance” and (antibiotic or antimicrobial or anti-bacterial or antibacterial) and “hospital pharmacy” and pharmacist and trial

* Automatic activated options in PubMed: randomized controlled trial and clinical trial.

**Table 2:** Main findings from the selected studies/trials.

Ref. Impact Factor JCR 2022 (or SJR if JCR Not Applicable) Year of Publication/Country	Design/Settings/Study Objectives	Pharmaceutical Intervention According to the Five Descriptors *	Methodology for Measuring Adherence or Compliance **	Control Group vs. Intervention (n° of Participants)	Main Findings and Conclusion
(Ngoh & Shepherd, 1997) [13] *Patient Educ Couns*. IF = 3.5 1997/Cameroon, West Africa	Controlled study/three health centers in Cameroon (pro pharmacy)/to compare comprehension and compliance with antibiotics in three groups	Groups: Control vs. Visual aids alone vs. Visual aids plus an Advanced Organizer (i.e., explanation about why the drug is needed) Descriptor: visual aid	c = 100 × [a − |a − b|]/a c = compliance expressed as a%; a = amount of medicine the patient should have taken if the prescriber’s instructions had been followed from the day treatment was started until the day of the researcher’s visit; b = [amount of medicine obtained by the patient from the propharmacy] minus [amount of medicine left in the container on the day of the researcher’s home visit]	Control (n = 26) vs. Two experimental groups (n = 26) (randomized)	The compliance and comprehension of nonliterate patients can be improved with statistical significance using visual aids (which was specifically produced) or a visual aid plus an advanced organizer.
(NMcMahon et al., 1997) [14] *Pediatrics.* IF = 5.1 1997/USA	Controlled study/general pediatric clinic/to determine whether parental errors in dosing liquid medication can be decreased through education	Group 1: prescription and verbal instructions Group 2: prescription and a syringe, with a demonstration of the correct dose Group 3: prescription, a syringe with a line marked at the correct dose and a demonstration Antibiotic suspension for otitis media Descriptor: dispensation of a syringe for correct dosing	At follow-up: parents were questioned about how much medication was administered, how many times a day, and for how many days.	Groups 1, 2 and 3 (n = 30) (randomized)	Group 1 (37% received the correct dose); Group 2 (83% received the correct dose) and Group 3 (100% received the correct dose). Medication dosing errors can be decreased with statistical significance through the use of a syringe.
(Al-Eidan et al., 2002) [15] *British Journal of Clinical Pharmacology.* IF = 5.8 2002/UK	Controlled study/hospital pharmacy/to evaluate the influence of patient counseling and follow-up on *H. pylori* eradication rates and to document the effectiveness of a 1-week eradication regimen	Intervention: counseling on disease, adverse drug reactions, and importance of compliance (and follow-up) by a hospital pharmacist. Control: standard advice sheet and referred to their GP. Descriptor: counseling in the case of *H. pylori* treatment	A questionnaire after completion of therapy (phone interviews) and pill count at the 4–6 weeks after.	Intervention group (n = 38); control group (n = 38). (randomized)	*H. pylori* eradication rate (94.7% intervention vs 73.7%; *p* = 0.02) and compliance (92.1% intervention vs. 23.7; *p* < 0.001). Structured patient counseling and follow-up can have a statistically significant effect on *H. pylori* eradication rates and should be a routine part of therapy.
(Stevens et al., 2002) [16] *The Western Journal of Medicine.* IF = 3.9 2002/USA	Controlled study/nonprofit group-practice health maintenance organization/to evaluate the eradication of *H. pylori* (3-month follow-up), to identity symptoms as reported on the symptom questionnaire and satisfaction with treatment and self-reported adherence to treatment	Usual-care counseling from a pharmacist for 5 min (control) or for 15 min and a follow-up phone call from the pharmacist during drug treatment (special-intervention group). Descriptor: counseling in the case of *H. pylori* treatment.	8 days after they started the medication (reported adherence by telephone)	Control (n = 154) Intervention (n = 148) (randomized)	% of participants missing one or more doses of each drug: 7.7%, 17.2%, 15.0%, and 16.6% (usual-care group), and 4.9%, 12.2%, 11.0%, and 12.2% (special- intervention group). Additional pharmacist counseling did not show an extra benefit in., *H. pylori* eradication, symptoms, and treatment adherence. However, both groups received counseling by a pharmacist and the % of self-reported missing doses was lower in the special-intervention group (without statistical significance) and patient satisfaction was better in the special-intervention group (with statistical significance).
(Beaucage et al., 2006) [17] *Am J Health-Syst Pharm.* IF = 2.7 2004/Canada	Controlled study/6 community pharmacies/to evaluate the impact in the community setting of a pharmacist telephone follow-up intervention in diverse clinical outcomes, such as adherence	Both groups (control and intervention): oral and written information on antibiotic dosage, the most significant potential adverse effects, and the importance of adherence to treatment. Intervention: additionally, patients received a telephone call from a pharmacist on day 3 of their antibiotic treatment. Descriptor: using both oral and written information in the intervention group	Number of antibiotic tablets or capsules left by patients (by phone).	Control (n= 129) and intervention (n = 126) (randomized)	Mean ± S.D. adherence to antibiotic: 94% ± 9% intervention and 94% ± 12% control groups (*p* = 0.803). An additional phone call slightly improved adherence, though without statistical significance.
(Eyler et al., 2016) [18] *The Consultant Pharmacist: The Journal of the American Society of Consultant Pharmacists.* H-INDEX SJR 25 2016/USA	Controlled study/medical wards in a large tertiary academic medical center/to evaluate the impact of a pharmacist-led, motivational interviewing on antibiotic adherence following discharge of older adults with pneumonia	Control: standard care Intervention: Motivational interviewing—a patient-centered method of communication—to address negative health behaviors (e.g., adherence) Descriptor: oral education-based interventions	Evaluation: (1) 24–48 h after discharge, retail pharmacies were contacted to check whether the antibiotic was picked up, and (2) in the last day of an antibiotic, a follow-up phone call	Control (n = 14) vs. Intervention (n = 16) (randomized)	% of antibiotic adherence: 87% (intervention) vs. 64% (control) (*p* = 0.14). Pharmacist-led motivational interviewing sessions presented a positive impact on patients’ antibiotic adherence, but without statistical significance.
(Treibich et al., 20217) [19] *PLoS ONE.* IF = 3.75 2017/France	Controlled study/community pharmacies (n = 100)/to assess the feasibility and the real impact of a change in the method of dispensing antibiotics in French community pharmacies for 14 antibiotics	Intervention: patients were asked by pharmacist if they agree with the supplying of antibiotics per unit (personalized delivery) Control: usual standard packaging Descriptor: personalized delivery (per unit)	Interview by phone two to three days (on average) after completion of their treatment	Control (n = 278) vs. Intervention (n = 907) (randomized)	65.6% (control) and 91.4% (intervention) of adherent patients (*p* < 0.00). The personalized delivery presented a positive and statistically significant impact on adherence and reduction in waste.
(West & Cordina, 2019) [20] *Research in Social & Administrative Pharmacy.* IF = 3.9 2019/Malta	Controlled Study/community pharmacies (n = 14)/to assess whether an intervention supported by an educational leaflet enhances adherence and reduces cost in relation to wastage of unused antibiotics and to determine a possible association between adherence and patients’ general medicines’ beliefs	Control: usual counseling; Intervention: usual counseling plus and educational leaflet/patients taking short-term antibiotics Descriptor: using both oral and written information in the intervention group	Contact by phone: the day following the stipulated termination date of an antibiotic course, patients were asked to count the amount of leftover antibiotic tablets/capsules	Control (n = 200) vs. Intervention (n = 200) (randomized)	Nonadherence: control (24%) vs. intervention (10%) (*p* ≤ 0.0005). An educational intervention (leaflet) significantly enhanced adherence and reduced wastage. Information about antibiotic resistances seems to have a positive impact on antibiotic adherence. Patients’ beliefs should be taken into consideration when counseling patients. The administration of a leaflet seems to support pharmacists’ structured counseling.
(Shoiab et al., 2023) [21] H-INDEX SJR35 2023/Jordan	Controlled study/hospital/to examine the impact of pharmacist counseling and follow-up on patients’ medication compliance and Helicobacter Pylori (*H. pylori*) eradication and evaluate the efficiency of an eradication regimen	Intervention: educational program on *H. pylori* infection and treatment plus follow-up 3 days after starting therapy Descriptor: counseling in the case of *H. pylori* treatment	The remaining pills were counted by researchers after the end of *H. pylori* treatment program	Control (n = 100) vs. Intervention (n = 100) (randomized)	Intervention vs. control: compliance 45.0% vs 27.5%; and eradication of *H. pylori* 28.5% vs 42.5% (*p* < 0.05, both). Pharmacist counseling improved with significant compliance and eradication of *H. pylori*. Thus, pharmacist counseling and follow-up should be practiced in regular clinical procedures.
(Almomani et al., 2023) [22] *PLoS ONE.* IF = 3.75 2023/Jordan	Controlled study/hospital/to evaluate the impact of an educational intervention on antibiotic short-term adherence and to assess the antibiotic utilization pattern	Intervention: education about the correct use of antibiotics Control: routine pharmaceutical care. Descriptor: oral education-based interventions	Phone call (two days after completing the antibiotics); questions: (i) any missing doses/days of the prescribed antibiotics and (ii) number of untaken/remaining pills (subjective vs. objective methods, respectively)	Control (n = 308) vs. Intervention (n = 304) (randomized)	Adherence to antibiotics in intervention (OR = 1.445, 95CI% = 1.029–2.030, *p* value = 0.033). Pharmacist intervention significantly enhanced adherence. The main motives for not taking antibiotics were observed improvement, forgetfulness about taking medication, and carelessness about taking the medication.
(Pham et al., 2013) [23] *SAGE Open Medicine.* IF = 2.3 2013/USA	Controlled study/2 community pharmacies/to evaluate whether medication counseling with emphasis on auxiliary labels improves recall of auxiliary label information and adherence to medication schedules	Intervention: written labels plus medication counseling based on three interview questions: “What did your doctor tell you the medication was for?”; “How did your doctor tell you to take this medication?”; “What did your doctor tell you to expect about your medication?” (10 to 15 min). Control: no medication counseling Follow-up: phone call (5 to 7 days after medication pickup) Descriptor: using both oral and written information in the intervention group	Phone call (5 to 7 days after medication pickup) to collect data on patient-reported assessments of adherence to the antibiotic schedule and duration of use	Control (n = 26) vs. Intervention (n = 24) (randomized)	Control: 7 of 21(33.3%) nonadherent. Intervention: 5 of 18 (27.8%) Nonadherent (*p* = 0.7). Pharmacists’ counseling has the potential to improve recall of information and adherence to antibiotics (without statistically significant differences). Counseling about dietary restrictions may need to be optimized (e.g., “Do not take dairy products, antacids or iron preparations within one hour of these medications”).
(Marques et al., 2023) [24] *Infectious Diseases Now.* IF = 3.5 2023/France	Controlled study/hospital/to assess the effectiveness of pharmacist-led-intervention (PLI) regarding six-month readmissions of patients with bone and joint infections	Intervention: standardized care plus PLI Control: standardized care PLI: pharmacist informed patients about antibiotic treatment and potential side effects Descriptor: oral education-based interventions	Telephone interview	Control (n = 105) vs. Intervention (n = 59)	There was a statistically significant positive impact of PLI on the reduction in 6-month readmissions, including less non-adherent patients. Example of causes of treatment modifications at six weeks and readmissions in six months: noncompliance (control 3.8% vs. intervention 1.6%).
(Göktay et al., 2013) [25] *Marmara Pharmaceutical Journal.* H-INDEX SJR 35 2013/Turkey	Controlled study/community pharmacy/to assess the impact of patient education on adherence	Education about antibiotic therapy: more comprehensive in intervention vs. brief education in control; only about the dosage regimen prescribed (both orally and written in the container) Descriptor: using both oral and written information in the intervention group	Two methodologies: self-report by patients and count of antibiotic tablets (the day after antibiotic therapy ended)	Control (n = 29) vs. Intervention (n = 31)	Patients in the intervention group were more adherent than those in the control group, although without statistically significant differences. Patients who were <30 years and were receiving multiple doses in the long term may benefit more from pharmacist education regarding antibiotic adherence.
(Merks et al., 2019) [26] *Patient Preference and Adherence.* IF = 2.2 2019/Poland	Controlled study/community pharmacy/to evaluate the practical utility of pharmaceutical pictograms in routine practice in a community pharmacy	Intervention: the antibiotic was dispensed with pictograms about drug regimen plus usual care Control: usual care Descriptor: visual aid	A short interview for both groups in a community pharmacy or by phone	Control (n = 102) vs. Intervention (n = 97) (randomized)	15.7% control vs. 13.4% intervention, discontinued therapy. Pictograms about a drug regimen can contribute to improving adherence to antibiotics (without statistical significance). Study pictograms were readily accepted by patients.
(Paravattil et al., 2021) [27] *Antibiotics.* IF = 4.8 2021/Qatar	Controlled study/community pharmacy/to evaluate the appropriateness of antibiotic prescriptions in community pharmacy settings while implementing an interventional call-back service to assess adherence and symptom resolution among patients prescribed an antibiotic	Call-back group: intensive antibiotic counseling and a phone call 3–5 days after antibiotic initiation Standard care: routine care—pharmacists from counseling and call-back attended training sessions Descriptor: oral education-based interventions	Adherence (1–2 days after the completion of an antibiotic): all patients were asked about the remaining tablets, symptom severity score, and satisfaction with the counseling	Standard care (n = 25), Counseling (n = 29), and Call-Back (n = 26) (randomized)	64% (standard care), 86.2% (Counseling), and 88.5% (Call-Back). Study intervention (intensive counseling or call-back) produced a positive and significant impact on adherence.
(Ormeci et al., 2015) [28] *Abstract European Journal of Hospital Pharmacy.* IF = 1.7 2015/Turkey	Controlled study/to evaluate the effect of patient education on compliance with prescribed antibacterial agents	Intervention: better informed and educated Control: basic information Descriptor: oral education-based interventions	One day after the end of the treatment: number of pills remaining in blister packs or containers, omitting the treatment, or missing a dose, at what time patient took the drugs, feeling better or not and whether the patient leaflet had been read	Control (n = 99) vs. Intervention (n = 100)	Conclusion: Intervention (more informed and educated) group presented higher compliance rates and improved clinical outcome, with statistical significance.
(Muñoz et al., 2014) [29] *Atención Primaria.* IF = 2.2 2014/Espanha	Controlled study/community pharmacy/to assess the effectiveness of an educational intervention on antibiotic adherence and patient-reported resolution of symptoms	Intervention: information on duration, dose, and method of use) and correct compliance (20 min) Control: routine care (questions/doubts were clarified) Telephone interview (one week after dispensation) Descriptor: oral education-based interventions	Two methodologies: Morisky---Green test and a self-reported pill count	Control (n = 62) vs. Intervention (n = 64) (randomized)	Adherence: Control 48.4% vs. 67.2% Intervention (*p* = 0.033). Non-compliance (missing more than one dose): Control 81.2% vs. 38.1% Intervention (*p* = 0.001). The educational intervention improved patients’ antibiotic adherence, with statistical significance. Medication knowledge was identified as a predictor of adherence.
(Jackson et al., 2006) [30] *Patient Educ Couns.* IF = 3.5 2006/UK	Controlled study/community pharmacy/to evaluate if implementation intentions increased adherence to short-term antibiotics in a patient sample	All groups were asked to take the antibiotic as prescribed Four groups: Control, Theory of Planned Behavior (TPB) questionnaire, TPB questionnaire + formed own implementation intention to take a medicine, e.g., “You are more likely to carry out your intention to take these antibiotics as prescribe if you make a decision about when and where you will do so” Descriptor: oral education-based interventions	Participants were asked about how many tablets were left (telephone interview after completing antibiotic treatment)	Control (n = 63) vs. TBP only group (n = 54) vs. TBP + own implementation intention − own (n = 53) vs. TBP + researcher implementation intention − given (n = 50) (randomized)	None tablet left: control (74.1%) vs. TBP only (78.4%) vs. TPB + own (73.1%) vs. TPB + given (78.3%). Implementation intentions did not improve antibiotic adherence, with statistical significance. An implementation intention strategy (by oneself or by the researcher) may be helpful for patients who report having forgotten to take their medication.
(Widowati et al., 2022) [31] *International Journal of Public Health Science.* H-INDEX SJR 35 2022/Indonesia	Controlled study/community pharmacy/to examine the effectiveness of the modified pharmacy counseling (MPC) model in improving short-term antibiotic compliance in outpatients	Baseline study: information on the level of knowledge and attitudes of the respondents on the short-term use of antibiotics Intervention: MPC model is a tool designed to help community pharmacists in developing their skills Follow-up (by telephone): Morisky medication adherence scale-8 (MMAS-8) questionnaire was conducted (3–7 days after antibiotic completion) Descriptor: oral education-based interventions	Morisky medication adherence scale-8 (at follow-up interview by telephone)	Control (n =144) vs. Intervention (n = 146) (randomized)	Compliance: 12.5% Control vs. 35.6% Intervention (*p* < 0.001). Compliance with a short-term antibiotic can be statistically improved through the MPC model. For instance, patient instructions should be simple, clear, and accompanied by a written version.
(González et al., 2003) [32] *Ars Pharmaceutica.* H-INDEX SJR 20 2003/Spain	Controlled study/community pharmacy/to evaluate the influence that written information has on compliance with antibiotic therapy, and to verify the consequences of degree of compliance on patient health	Both received the same information: dosage schedules and the duration of the treatment, as well as the lifestyle habits that would benefit the cure for their infections The intervention also received a reinforcement (written information) Descriptor: using both oral and written information in the intervention group	Telephone interview; the day after finishing the treatment	Control (n = 109) vs. Intervention (n = 105) (randomized)	Compliance: 46.8% Control vs. 61% Intervention (*p* = 0.038). Written information improved, with statistically significant patient compliance and patients’ perceptions of health.
(Gotsch et al., 1982) [33] *Medical Care.* IF = 3 1982/USA	Controlled study/community pharmacy/To measure the effectiveness of patient package inserts (PPIs) when controlled interventions by pharmacists are increased	Educational intervention I (control): pharmacists responded to any questions patients, but other oral or written information was not provided Educational Intervention II: reply to any question + PPI Educational Intervention III: reply to any question + PPI + reinforcement of information by the pharmacist Descriptor: using both oral and written information in the intervention group	Telephone interview (not more than 3 days before the end of antibiotic therapy); the number of remaining doses were counted by participants	Educational Intervention I (control) (n = 62) Educational Intervention II (n = 62) Educational Intervention III (n = 62) (not randomized)	Noncompliance: 53% control vs. 43% intervention I vs. 28% intervention II. Compliance can be enhanced through the administration of PPI, with statistical significance especially when information is verbally reinforced by a pharmacist.

* The five descriptors are as follows: (i) visual aid [13,26], (ii) dispensation of a syringe for correct dosing or personalized delivery (per unit) [14,19]; (iii) using both oral and written information in the intervention group [17,20,23,25,32,33]; (iv) oral education-based interventions (excluding the counseling in the case of *H. pylori* treatment) [18,22,24,27,28,29,30,31], and (v) counseling in the case of *H. pylori* treatment [15,16,21] since these therapeutics usually involve multiple medicines, which may complicate patient adherence. ** The terms adherence and compliance were used as synonyms in some of the selected studies.

**Table 3:** % of compliant assessments according to the NHLBI tool [34].

Question	%
1. Was the study described as randomized, a randomized trial, a randomized clinical trial, or an RCT?	89.5
2. Was the method of randomization adequate (i.e., the use of randomly generated assignment)?	47.4
3. Was the treatment allocation concealed (so that assignments could not be predicted)?	26.3
4. Were study participants and providers blinded to treatment group assignment?	n.a.
5. Were the people assessing the outcomes blinded to the participants’ group assignments?	21.1
6. Were the groups similar at the baseline in important characteristics that could have affected outcomes (e.g., demographics, risk factors, co-morbid conditions)?	84.2
7. Was the overall drop-out rate from the study at the endpoint 20% or lower than the number allocated to treatment?	89.5
8. Was the differential drop-out rate (between treatment groups) at the endpoint 15 percentage points or lower?	84.2
9. Was there high adherence to the intervention protocols for each treatment group?	100
10. Were other interventions avoided or similar in the groups (e.g., similar background treatments)?	100
11. Were outcomes assessed using valid and reliable measures that were implemented consistently across all study participants?	100
12. Did the authors report that the sample size was sufficiently large to be able to detect a difference in the main outcome between groups with at least 80% power?	52.6
13. Were the outcomes reported or subgroups analyzed prespecified (i.e., identified before analyses were conducted)?	100
14. Were all randomized participants analyzed in the group to which they were originally assigned, i.e., did the authors use an intention-to-treat analysis?	10.5

n.a.: question classified as not applicable (please see the methods described in Section 2.9).

**Table 4:** GRADE-CERQual summary.

Summary of Review/Finding	References	CERQual Assessment of Confidence	Explanation of CERQual Assessment
The pharmacist intervention improved patients’ adherence to antibiotics. For instance, the provision of a leaflet/package insert and/or pharmacist counseling/advice/education, the intention to take the antibiotic, a follow-up phone call, a motivational interview, guided advice (model-based), or the personalized delivery of antibiotics.	[15,16,17,18,19,20,22,29,30,31,32] (n = 11 trials out of 21; 52.4%)	High	The pharmacist intervention improved patients’ adherence to antibiotics. For instance, the provision of a leaflet/package insert and/or pharmacist counseling/advice/education, the intention to take the antibiotic, a follow-up phone call, a motivational interview, guided advice (model-based), or the personalized delivery of antibiotics.
The pharmacist intervention improved patients’ adherence to antibiotics. For instance, visual aid/pictograms, the dispensation of a syringe with the demonstration of the correct dose, the provision of written information/a package insert, interview questions, pharmacist counseling/advice/education, and/or a call back/phone call.	[13,14,21,23,24,25,26,27,28,33] (n = 10 trial out of 21; 47.6%)	Moderate	Concerns related to the relevance, adequacy, and coherence of the data were identified. Potential methodological issues were detected (only the published papers were consulted, i.e., full protocols were not available for public consultation).

## Data Availability

The original contributions presented in this study are included in the article. Further inquiries can be directed to the corresponding author.
